# A novel semi-supervised model for miRNA-disease association prediction based on $\ell_{1}$-norm graph

**DOI:** 10.1186/s12967-018-1741-y

**Published:** 2018-12-14

**Authors:** Cheng Liang, Shengpeng Yu, Ka-Chun Wong, Jiawei Luo

**Affiliations:** 1 School of Information Science and Engineering, Shandong Normal University, Jinan, 250358 China; 2 Department of Computer Science, City University of Hong Kong, Kowloon Tong, 999077 Hong Kong; 3 College of Information Science and Engineering, Hunan University, Changsha, 410082 China

**Keywords:** miRNA-disease association, $\ell_{1}$-norm graph, Semi-supervised learning

## Abstract

**Background:**

Identification of miRNA-disease associations has attracted much attention recently due to the functional roles of miRNAs implicated in various biological and pathological processes. Great efforts have been made to discover the potential associations between miRNAs and diseases both experimentally and computationally. Although reliable, the experimental methods are in general time-consuming and labor-intensive. In comparison, computational methods are more efficient and applicable to large-scale datasets.

**Methods:**

In this paper, we propose a novel semi-supervised model to predict miRNA-disease associations via $\ell_{1}$-norm graph. Specifically, we first recalculate the miRNA functional similarities as well as the disease semantic similarities based on the latest version of MeSH descriptors and HMDD. We then update the similarity matrices and association matrix iteratively in both miRNA space and disease space. The optimized association matrices from each space are combined together as the final output.

**Results:**

Compared with four state-of-the-art prediction methods, our method achieved favorable performance with AUCs of 0.943 and 0.946 in both global LOOCV and local LOOCV, respectively. In addition, we carried out three types of case studies on five common human diseases, and most of the top 50 predicted miRNAs were confirmed to be associated with the investigated diseases by four databases dbDEMC, PheomiR, miR2Disease and miRwayDB. Specifically, our results provided potential evidence that miRNAs within the same family or cluster were likely to play functional roles together in given diseases.

**Conclusions:**

Taken together, the experimental results clearly demonstrated the utility of the proposed method. We anticipated that our method could serve as a reliable and efficient tool for miRNA-disease association prediction.

**Electronic supplementary material:**

The online version of this article (10.1186/s12967-018-1741-y) contains supplementary material, which is available to authorized users.

## Background

MicroRNAs (miRNAs) are small single-stranded RNAs that repress mRNA translation and trigger mRNA degradation at the post-transcriptional level. Since the discovery of the first two miRNAs in mammalian cells, there has been a tremendous and growing interest among researchers to investigate the role of miRNAs in normal cellular as well as the disease processes [1]. Compelling evidence have demonstrated the fundamental importance of miRNAs in normal development, differentiation, growth control and in human diseases such as cancer [2]. For instance, the overexpression of miR-193a-3p and miR-224 increases cell proliferation in renal cell carcinoma by directly targeting ST3GallV via PI3K/Akt pathway [3], and miR-197 induces epithelial–mesenchymal transition and invasion through the downregulation of HIPK2 in lung adenocarcinoma [4]. Emerging evidence also suggested that substitution of tumor suppressive miRNAs or inhibition of oncogenic miRNAs could be used to develop novel treatment strategies [5]. Therefore, the identification of the disease-related miRNAs is of great significance for the new drug design and therapeutic development for complex human diseases.

Great efforts have been made to discover potential associations between miRNAs and diseases using experimental approaches. Jones et al. found that miR-186-5p was involved in the prostate cancer cell proliferation and invasion through qRT-PCR and western blot [6]. Similarly, Cui et al. found that the decreased miR-337 expression was significantly associated with tumor stage and lymph node metastasis of hepatocellular carcinoma based on the analysis of transfection of miR-337 mimics [7]. Although reliable, experimental methods are generally time-consuming and cannot be applied to large-scale datasets. With the accumulation of multiple data sources, a number of computational methods have been developed to predict reliable miRNA-disease associations [8–10]. Under the assumption that functionally related miRNAs tend to be involved in phenotypically similar diseases and vice versa, Jiang et al. developed the first computational model to prioritize the disease-related miRNAs by constructing a scoring system based on hypergeometric distribution [11]. Following their seminal work, Chen et al adopted global network similarities and developed random walk with restart to infer potential miRNA-disease associations [12]. Shi et al. also used the random walk with restart to calculate an enrichment score by integrating the miRNA target information as well as the protein–protein interactions [13]. Xuan et al. first calculated the miRNA functional similarity by taking miRNA family and cluster information into account, and then prioritized disease-related miRNAs in terms of the weighted *k* most similar neighbors [14]. However, their method cannot be applied to diseases without any known associated miRNAs. To solve this issue, they proposed another approach called MIDPE based on bilayer random walk model later on, in which different categories of nodes were assigned different transition weights [15]. Mørk et al. inferred the miRNA-disease associations by coupling known and predicted miRNA-protein associations with protein-disease associations text mined from the literature. Besides linking miRNAs to diseases, it directly suggested the underlying proteins that can be further validated experimentally [16]. By taking advantage of tissue-specific miRNA expression profiles and miRNA target information, Zhao et al. calculated the enrichment significance of the known pathway over gene clusters to identify cancer-related miRNAs [17]. Nonetheless, their method relies on tissue-specific miRNA expression profiles, which might be difficult to obtain sometimes. Chen et al. first calculated the within-score and between-score from the view of miRNA and diseases respectively, and then combined them together to obtain final scores for the prioritization of the miRNA-disease associations [18]. Liu et al. implemented random walk on a heterogeneous network which was constructed by integrating multiple data sources, including gene functional similarities, miRNA-target gene associations, miRNA-lncRNA associations, lncRNA similarity and etc., which improved the prediction accuracy of previous methods [19]. Recently, Chen et al. proposed Heterogeneous Graph Inference for MiRNA-Disease Association (HGIMDA) by iteratively updating the association matrix based on the miRNA functional similarity matrix and disease semantic similarity simultaneously [20]. The leave-one-out cross validation demonstrated that HGIMDA achieved comparable results.

Several machine learning-based models were also developed to predict potential miRNA-disease associations. Jiang et al. extracted a set of features for each positive and negative miRNA-disease association and trained a support vector machine (SVM) for the classification [21]. Chen et al. constructed a continuous classification function based on regularized least squares to reflect the probability of each miRNA related to a given disease [22]. Pasquier et al. represented distributional information on miRNAs and diseases in a high-dimensional vector space and the miRNA-disease association scores were calculated in terms of their vector similarity [23]. Shen et al. developed a computational method based on collaborative matrix factorization for miRNA-disease association prediction by integrating miRNA functional similarity, disease semantic similarity and known miRNA-disease associations [24]. Luo et al. developed a collective prediction model based on transductive learning to systematically prioritize disease-related miRNAs. They calculated a relevance score for each association and updated the network structure iteratively until convergence [25]. Chen et al. presented a novel computational model called MKRMDA in which Kronecker regularized least squares were calculated based on multiple kernels for miRNA-disease association prediction [26]. However, there were several parameters involved in their model and how to appropriately choose proper values is not a trivial task. They further proposed a model of Extreme Gradient Boosting Machine for MiRNA-Disease Association (EGBMMDA). For each miRNA-disease pair, they formed an informative feature vector by combining results obtained from statistical measures, graph theoretical measures and matrix factorization results. The feature vector was then used to train a regression tree under the gradient boosting framework [27]. Recently, Fu and Peng proposed a deep ensemble model called DeepMDA which extracts high-level features from similarity information using stacked autoencoders [28]. The miRNA-disease associations were then predicted based on a three-layer neural network. Xiao et al. presented a graph regularized non-negative matrix factorization method for identifying miRNA-disease associations. Experiment results indicated that their method could effectively prioritize disease-associated miRNAs with higher accuracy compared with other alternatives [29].

Another family of methods considers the network topology when predicting miRNA-disease associations. Sun et al. presented a computational method named NTSMDA that utilized the known miRNA-disease network topological similarity to exploit potential disease-related miRNAs [30]. You et al. proposed a Path-Based MiRNA-Disease Association (PBMDA) prediction model. They first constructed a heterogeneous graph consisting of three interlinked sub-graphs and then used depth-first algorithm to infer potential miRNA-disease associations [31]. However, the maximum length of paths cannot be larger than four due to the exponential computational complexity. Chen et al. developed a computational model named NDAMDA that not only considered the direct network distance between two miRNAs or diseases but also took their respective mean network distances to all other miRNAs or diseases into account [32]. They further proposed to use the graphlet interaction to analyze the complex relationships between miRNA or disease pairs in a graph. Specifically, they counted the number of different graphlet interaction isomers to calculate relevance scores for miRNA-disease associations. Nevertheless, their method cannot scale to graphlets that contain more than four nodes [33].

Although existing methods have achieved remarkable performances, there are still some limitations to be solved. Briefly, due to the intrinsic noise as well as the incompleteness existing in the current datasets, it is difficult to obtain reliable similarity matrices for both miRNAs and diseases. Moreover, the fact that no true negative datasets were validated might influence the prediction performance of the machine learning-based methods. Consequently, how to predict miRNA-disease associations reliably and effectively still remains a challenging task. To solve the above problems, in this paper, we first recalculate the similarity matrices for both miRNAs and diseases with the latest version of Mesh database (http://www.ncbi.nlm.nih.gov/) and HMDD [34]; we then propose a novel semi-supervised prediction method based on $\ell_{1}$-norm graph model. Specifically, both miRNA and disease similarity matrices could be adaptively re-weighted during the iteration process and the label matrix could be updated accordingly. To demonstrate the effectiveness of our method, we apply global leave-one-out cross validation (global LOOCV) and local leave-one-out cross validation (local LOOCV) to evaluate the prediction performance. The experiment results show that our method achieved AUCs of 0.943 and 0.946 for global LOOCV and local LOOCV, respectively. The case studies on five common human diseases further confirm the utility of our method. Together, we present a novel framework for miRNA-disease association prediction and envision it being a useful tool for future clinical analysis.

## Methods

### Disease semantic similarity

According to the previous study [35], we downloaded the latest MeSH descriptors from the National Library of Medicine (http://www.nlm.nih.gov/) and only kept the items from Category C for diseases, which resulted in 11,572 unique items. As described in [35], the relationship among different diseases can be represented as a Directed Acyclic Graph (DAG). For a given disease *d*, its DAG can be denoted as *DAG *= (*d*, *T*(*d*), *E*(*d*)), where *T*(*d*) represents all the ancestor nodes of *d* and *d* itself, and *E*(*d*) represents all direct edges connecting the parent nodes to child nodes. The contribution *D*_*d*_(*t*) of a disease *t* in *DAG*_*d*_ to the semantics of disease *d* could be calculated by:

1 $$\left\{ \begin{aligned} & D_{d} \left( d \right) = 1 \\ & D_{d} \left( t \right) = max\left\{ {0.5\,*\,D_{d} \left( {t^{\prime}} \right)|t^{\prime} \in children \,of\, t} \right\} \quad if\,t \ne d \\ \end{aligned} \right.$$

Based on Eq. (1), the semantic value *DV* of a given disease *d* could be defined as follows:

2 $$DV\left( d \right) = \mathop \sum \limits_{t \in T\left( d \right)} D_{d} \left( t \right)$$

Apparently, diseases with more common items will have greater semantic similarities. Finally, the semantic similarity score between two diseases *i* and *j* is defined as follows:

3 $$S\left( {i,j} \right) = \frac{{\mathop \sum \nolimits_{t \in T\left( i \right)\mathop \cap \nolimits T\left( j \right)} \left( {D_{i} \left( t \right) + D_{j} \left( t \right)} \right)}}{DV\left( i \right) + DV\left( j \right)}$$

Moreover, the similarity of a given disease *d* and a group of diseases $D_{t} = \left\{ {d_{t1} , d_{t2} , \ldots , d_{tk} } \right\}$ was defined by:

4 $$S\left( {d, D_{t} } \right) = \mathop {max}\limits_{{1 \le i \le k\left( {S\left( {d,d_{{ti}} } \right)} \right)}}$$

By using Eq. (3), we could obtain the semantic similarities for each disease pair. For convenience, we denote the disease semantic similarity matrix as *W*^*d*^, where the entity *W*^*d*^(*i*, *j*) represents the semantic similarity between disease *i* and disease *j*. The computed disease similarity matrix was provided in Additional file 1.

### Human miRNA-disease association data

The latest version of human miRNA-disease association data (v2.0) was downloaded from HMDD [34]. Besides, we also downloaded the latest version of existing miRNAs that was released on March 2018 from miRBase [36], which record 4796 human miRNAs. To keep consistent of data from different sources and eliminate as many false positives as possible, associations with miRNAs and diseases that were not recorded in miRBase and MeSH were excluded [37]. As a result, 6088 associations between 550 miRNAs and 328 diseases were used in the subsequent analysis (Additional file 2). Adjacency matrix *A* is adopted to represent the miRNA-disease associations. For a given miRNA *i* and disease *j*, *A*(*i*, *j*) = 1 if *i* is related to *j*, and *A*(*i*, *j*) = 0 otherwise.

### MiRNA functional similarity

To calculate the functional similarity between two miRNAs *M*1 and *M*2, we need to measure the contributions from similar diseases that are associated with both of them [35]. Let *DT*_1_ and *DT*_2_ represent the related diseases of miRNA *M*1 and *M*2, respectively. The functional similarity of *M*1 and *M*2 is then calculated as follows:

5 $${\text{MISIM}}\left( {M1, M2} \right) = \frac{{\mathop \sum \nolimits_{{1 \le i \le \left| {DT_{1} } \right|}} {\text{S}}\left( {dt_{1i} ,DT_{2} } \right) + \mathop \sum \nolimits_{{1 \le j \le \left| {DT_{2} } \right|}} {\text{S}}\left( {dt_{2j} ,DT_{1} } \right)}}{{\left| {DT_{1} \left| + \right|DT_{2} } \right|}}$$

where S(*dt*, *DT*) measures the similarity of a given disease *dt* and a set of diseases *DT* and its definition is given in Eq. (4). We use *W*^*m*^ to denote the miRNA functional similarity matrix, where the entity *W*^*m*^(*i*, *j*) represents the functional similarity between miRNA *i* and miRNA *j*. The computed miRNA similarity matrix was provided in Additional file 3.

### The proposed method

**Fig. 1:**
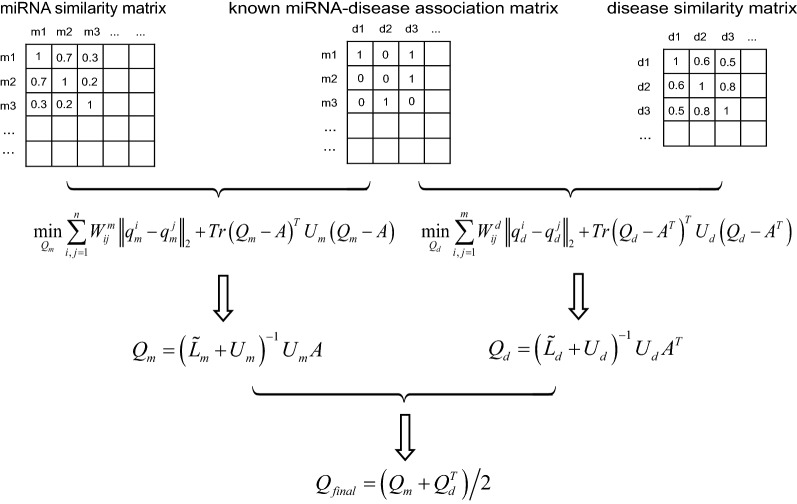
An overall workflow of the proposed method

To effectively predict the potential miRNA-disease associations, we here propose a novel semi-supervised method based on $\ell_{1}$-norm graph model (Fig. 1). Let *n* and *m* denote the number of miRNAs and diseases in our dataset, respectively. The dimension of the known association matrix *A* is thus *n *× *m*. Let us first consider the miRNA space. Given the association matrix *A* as well as the miRNA functional similarity matrix *W*^*m*^, our goal is to obtain an indicator matrix *Q*_*m*_ $\in {\mathbb{R}}^{n \times m}$ that could reflect the association probability between certain miRNAs and diseases. Since the solution to the traditional graph based semi-supervised learning is sensitive to noise and outliers [38, 39], we define the $\ell_{1}$-norm-based objective as follows:

6 $$\mathop {\hbox{min} }\limits_{{Q_{m} }} \sum\limits_{i,j = 1}^{n} {W_{ij}^{m} } \left\| {q_{m}^{i} - q_{m}^{j} } \right\|_{2} \, + \,Tr\left( {Q_{m} - A} \right)^{T} U_{m} \left( {Q_{m} \, - \,A} \right)$$

where $q_{m}^{i}$ and $q_{m}^{j}$ represent the *i*-th and *j*-th column of *Q*_*m*_, respectively. *U*_*m*_ is a diagonal matrix with the *i*-th diagonal element to control the impact of the initial associations from *A*.

Let *p*_*m*_ denote a *n*^2^-dimensional vector of which the ((*i *− 1)*n + *j*)-th element is $W_{ij}^{m} \left\| {q_{m}^{i} - q_{m}^{j} } \right\|_{2}$, we can re-write Eq. (6) as

7 $$\mathop {\hbox{min} }\limits_{{Q_{m} }} \left\| {p_{m} } \right\|_{1} \, + \,Tr\left( {Q_{m} - A} \right)^{T} U_{m} \left( {Q_{m} \, - \,A} \right)$$

which gives us the $\ell_{1}$-norm representation of our objective function. It is widely known that the $\ell_{1}$-norm usually generates sparse solutions and thus the solution to Eq. (7) will provide a more confident prediction results for potential miRNA-disease associations [40]. However, Eq. (7) is non-smooth and difficult to be solved efficiently [41]. To overcome this issue, we further defined a re-weighted similarity matrix as follows:

8 $$\tilde{W}_{ij}^{m} \, = \,\frac{{W_{ij}^{m} }}{{2\left\| {q_{m}^{i} \, - \,q_{m}^{j} } \right\|_{2} }}$$

where the similarity matrix *W*^*m*^ can be updated during each iteration. By integrating Eq. (8) into Eq. (6) and taking the derivative of Eq. (6) with respect to *Q*_*m*_, we have:

9 $$\begin{aligned} \tilde{L}_{m} Q_{m} \, + \,U_{m} \left( {Q_{m} \, - \,A} \right)\, = \,0 \hfill \\ \Rightarrow Q_{m} \, = \,\left( {\tilde{L}_{m} \, + \,U_{m} } \right)^{ - 1} U_{m} A \hfill \\ \end{aligned}$$

where $\tilde{L}_{m} \, = \,\tilde{D}_{m} \, - \,\tilde{W}^{m}$ is the Laplacian matrix and $\tilde{D}_{m}$ is a diagonal matrix with the *i*-th diagonal element as $\sum\nolimits_{j} {\tilde{W}_{ij}^{m} }$. Note that $\tilde{L}_{m}$ is dependent on $\tilde{W}^{m}$, we develop an iterative algorithm to solve *Q*_*m*_ until convergence. Similarly, we define the $\ell_{1}$-norm based objective for the disease space as follows:

10 $$\mathop {\hbox{min} }\limits_{{Q_{d} }} \sum\limits_{i,j = 1}^{m} {W_{ij}^{d} } \left\| {q_{d}^{i} - q_{d}^{j} } \right\|_{2} \, + \,Tr\left( {Q_{d} - A^{T} } \right)^{T} U_{d} \left( {Q_{d} \, - \,A^{T} } \right)$$

where *Q*_*d*_ $\in {\mathbb{R}}^{m \times n}$ is the label matrix to be solved. Following the same procedure presented above, we could obtain:

11 $$Q_{d} \, = \,\left( {\tilde{L}_{d} \, + \,U_{d} } \right)^{ - 1} U_{d} A^{T}$$

Combining Eq. (9) with Eq. (11), we could obtain the final prediction result *Q*_*final*_:

12 $$Q_{final} \, = \,{{\left( {Q_{m} \, + \,Q_{d}^{T} } \right)} \mathord{\left/ {\vphantom {{\left( {Q_{m} \, + \,Q_{d}^{T} } \right)} 2}} \right. \kern-0pt} 2}$$

The procedure of the proposed method is summarized in Algorithm 1. According to previous literature [38], Algorithm 1 is guaranteed to converge to the global optimum of the problem.
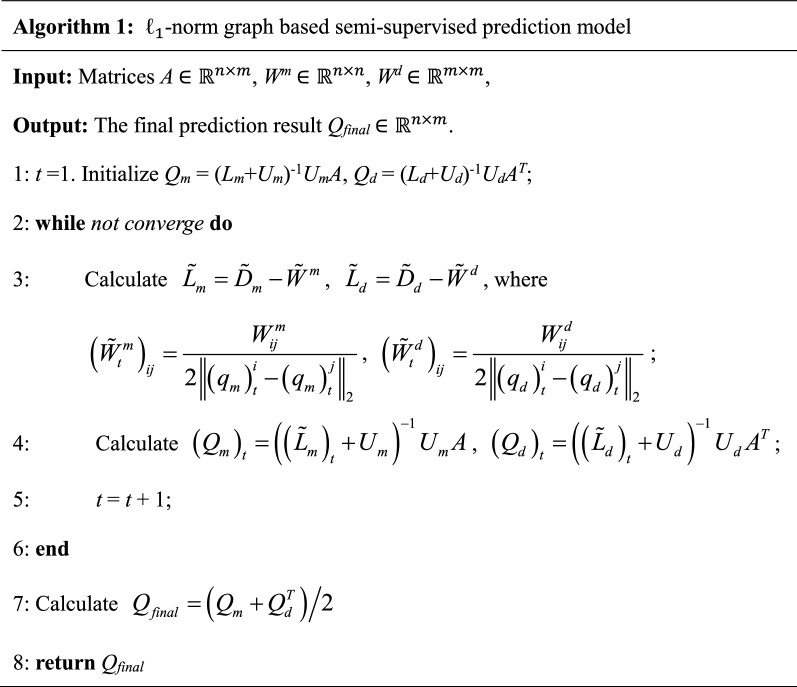


## Results

### Performance evaluation

**Fig. 2:**
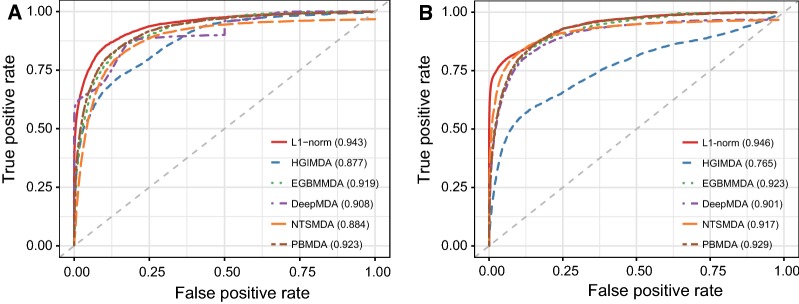
Comparison results between our method and the other five prediction methods in terms of (**a**) global LOOCV and (**b**) local LOOCV

To validate the prediction ability of our method, we implemented leave-one-out cross validation (LOOCV) where each known association was left in turn as the test sample and the rest of the known associations were used for optimization. LOOCV can be conducted in two ways, i.e. global LOOCV and local LOOCV. In global LOOCV, the test sample was ranked with all the other unconfirmed miRNA-disease associations, whereas in local LOOCV the test sample was ranked with all the unconfirmed associations of a given disease. Test samples with predicted values higher than a given threshold were considered as successful predictions. To intuitively evaluate the prediction performance, we adopted receiver operating characteristics (ROC) curve and calculated the area under the ROC curve (AUC). The larger the AUC, the better the prediction performance. Moreover, we compared our method with five state-of-the-art approaches, i.e. HGIMDA [20], EGBMMDA [27], DeepMDA [28], NTSMDA [30] and PBMDA [31]. As mentioned before, HGIMDA was an efficient prediction framework based on heterogeneous graph inference. EGBMMDA was an effective classification method based on extreme gradient boosting machine while DeepMDA was a deep ensemble classification model. Both NTSMDA and PBMDA took advantage of different network topological characteristics to prioritize disease-related miRNAs. The experimental results were demonstrated in Fig. 2. As a result, HGIMDA, EGBMMDA, DeepMDA, NTSMDA and PBMDA obtained AUCs of 0.877, 0.919, 0.908, 0.884 and 0.923 in global LOOCV, respectively. For local LOOCV, the five methods also obtained comparable AUCs of 0.765, 0.923, 0.901, 0.917 and 0.929, respectively. Notably, our method achieved the highest AUCs of 0.943 and 0.946 in both global LOOCV and local LOOCV, which clearly demonstrated the superior performance of our method in predicting potential miRNA-disease associations. In addition, we calculated the statistical significance of performance improvement gained by our method over the other methods to further validate the effectiveness of our method. Specifically, we first computed an AUC value for each disease and obtained a vector consisting of 328 AUC values for each method. We then assessed the statistical significance of difference between AUC values by Wilcoxon signed rank test. As shown in Table 1, our method significantly improved the prediction performance with respect to the other five methods.

**Table 1:** Statistical significance of difference in performance between the proposed method and the other five methods

	HGIMDA	EGBMMDA	DeepMDA	NTSMDA	PBMDA
*P*-value	1.59e−46	1.21e−29	2.43e−27	2.42e−25	3.24e−23

*P*-values were calculated by Wilcoxon signed rank test

We next examined the computational cost of all methods by evaluating their computational time and memory needed for each run. Experiments were performed on a computer cluster where each node is equipped with 2 AMD Dual-Core Opteron 8218 processors and 16 GB memory. As shown in Table 2, our method could achieve superior performance with a reasonable amount of computational resources.

**Table 2:** Computational time and memory needed for each run of all methods

	$\ell_{1}$-norm	HGIMDA	EGBMMDA	DeepMDA	NTSMDA	PBMDA
Programming Language	R	MATLAB	R	Python 2.7	R	Python 2.7
Time (min)	< 3	< 1	< 1	< 10	< 1	< 60
Memory (GB)	4	4	4	12	4	16

### Case studies

To further demonstrate the prediction ability of the proposed method, we carried out three types of case studies on five common diseases. Four databases dbDEMC [42], PhenomiR [43], miR2Disease [44] and miRwayDB [45] were used to validate the prediction results in all five case studies. Specifically, dbDEMC is an integrated database that records differentially expressed miRNAs in human cancers detected by high-throughput method, while PhenomiR, miR2Disease and miRwayDB provide information about differentially regulated miRNA expression in diseases and other biological processes or pathways completely generated by manual curation of experienced annotators. Since the miRNAs recorded in dbDEMC, miR2Disease as well as miRwayDB are annotated in their mature sequence form, we matched the candidate miRNAs with those recorded in the three aforementioned databases according to the miRNA nomenclature provided from miRBase. Besides, to validate our case study results across all the four databases, we selected 16 common diseases among them for the subsequent analysis. Due to space limitations, we only provided the validation results of five diseases here and the results of the other diseases can be found in additional files. For the first type of case studies, we applied our method to predict the potential associations between miRNAs and three given diseases, i.e. Lung Neoplasms, Ovarian Neoplasms and Prostatic Neoplasms based on the known associations in HMDD v2.0 (Additional file 4).

Lung cancer is the leading cause of cancer death among men and women worldwide, with an incidence of over 200,000 new cases per year coupled with a very high mortality rate [46]. Great efforts have been made to investigate the functional roles of miRNAs in lung cancer cell progression and resistance to therapy. For instance, recent studies have identified that miR-15a-3p could induce apoptosis in lung cancer cell lines and thus serve as a potential biomarker for apoptosis-modulating therapies in lung cancer treatment [47]. However, promising findings of a lung cancer-associated miRNAs in one study is inadequate to support a solid report, more studies would be needed to cross validate the discovery. Here, we carried out our first case study on this lethal disease and prioritized the top 50 ranked miRNAs by our method. As shown in Table 3, 49 out of the 50 predicted miRNAs were confirmed by experimental findings recorded in at least one of the four databases dbDEMC, PhenomiR, miR2Disease and miRwayDB. Specifically, three of the top four predicted miRNAs (i.e. hsa-mir-16-1, hsa-mir-16-2 and hsa-mir-15) were validated by all the databases. The only unconfirmed miRNA was hsa-mir-520b. Intriguingly, we observed that other miRNAs (i.e. hsa-mir-520d, hsa-mir-520c and hsa-520a) within the same miRNA family of hsa-mir-520b were all confirmed by dbDEMC. Therefore, hsa-mir-520b might also function as a potential regulator in the tumorigenesis and progression of lung cancer.

**Table 3:** Top 50 predicted miRNAs associated with lung neoplasms based on known associations in HMDD

Rank	miRNA	Evidence	Rank	miRNA	Evidence
1	hsa-mir-125b-2	I; II; III	26	hsa-mir-328	I; II
2	hsa-mir-16-1	I; II; III; IV	27	hsa-mir-122	I; II; IV
3	hsa-mir-16-2	I; II; III; IV	28	hsa-mir-424	I; II
4	hsa-mir-15a	I; II; III; IV	29	hsa-mir-520d	I
5	hsa-mir-199a-2	I; II	30	hsa-mir-99a	I; II; III; IV
6	hsa-mir-218-1	I; II; III	31	hsa-mir-449a	I; II
7	hsa-mir-451a	I	32	hsa-mir-302a	I; II
8	hsa-mir-106b	I; II	33	hsa-mir-302b	I; II
9	hsa-mir-92a-2	I; II	34	hsa-mir-342	I; II
10	hsa-mir-133a-2	I; II	35	hsa-mir-151b	I
11	hsa-mir-15b	I; II; III	36	hsa-mir-449b	I; II
12	hsa-mir-378a	I	37	hsa-mir-152	I; II
13	hsa-mir-193b	I; II	38	hsa-mir-485	I; II
14	hsa-mir-130a	I; II; III	39	hsa-mir-345	I; II; III
15	hsa-mir-429	I; III	40	hsa-mir-373	I; II
16	hsa-mir-141	I; II; III; IV	41	hsa-mir-204	I; II; III
17	hsa-mir-151a	I	42	hsa-mir-302c	I; II
18	hsa-mir-19b-2	I; II	43	hsa-mir-144	I; II; IV
19	hsa-mir-708	I; IV	44	hsa-mir-520c	I
20	hsa-mir-10a	I; II; IV	45	hsa-mir-194-1	I; II; IV
21	hsa-mir-149	I; II	46	hsa-mir-296	I; II
22	hsa-mir-195	I; II; III	47	hsa-mir-23b	I; II
23	hsa-mir-20b	I; II	48	hsa-mir-520a	I
24	hsa-mir-24-1	I; II	49	hsa-mir-520b	Unconfirmed
25	hsa-mir-625	I	50	hsa-mir-28	I; II

I, II, III and IV represent dbDEMC, PhenomiR, miR2Disease and miRwayDB, respectively

Ovarian neoplasms is the fifth most common cause of cancer deaths in women and has the highest mortality rate among all the gynecological malignancies. Its lethality is largely due to the difficulties in detecting it at an early stage and lack of effective treatments for patients with an advanced or recurrent status [48, 49]. Consequently, there is an urgent need to identify prognostic and predictive markers for early detection. Various miRNAs such as miR-200 family and let-7 paralogs have been proposed as potential therapeutic targets for disseminated or chemoresistant ovarian tumors. We implemented our method to prioritize the candidate miRNAs for ovarian neoplasms and the top 50 predicted miRNAs are given in Table 4. Similarly, 49 out of the 50 predicted miRNAs were confirmed by at least one databases from dbDEMC, PhenomiR, miR2Disease and miRwayDB. The only unconfirmed miRNA was hsa-mir-181a-2. As a matter of fact, in vivo experiments have implicated that miR-181a could modulate TGF-*β* signaling to induce and maintain epithelial–mesenchymal transition and further affect ovarian cancer cell survival [50]. In addition, three miRNAs (hsa-mir-181a-1, hsa-mir-181b-1 and hsa-mir-181b-2) from the same miRNA family of hsa-mir-181a-2 were all supported to be associated with ovarian cancer by dbDEMC. Together, our prediction provided new evidence for its association with ovarian cancer.

**Table 4:** Top 50 predicted miRNAs associated with ovarian neoplasms based on known associations in HMDD

Rank	miRNA	Evidence	Rank	miRNA	Evidence
1	hsa-mir-143	I; II; III	26	hsa-mir-124-3	I; II
2	hsa-mir-29c	I; II; III	27	hsa-mir-181b-2	I
3	hsa-mir-222	I; II	28	hsa-mir-24-1	I
4	hsa-mir-15a	I; II	29	hsa-mir-107	I; II
5	hsa-mir-210	I; II	30	hsa-mir-196a-1	I
6	hsa-mir-205	I; II	31	hsa-mir-26a-2	I; III
7	hsa-mir-142	I; II; III	32	hsa-mir-26a-1	I; II; III
8	hsa-mir-181a-1	I; II	33	hsa-mir-27b	I
9	hsa-mir-9-2	I; II; III; IV	34	hsa-mir-106a	I; II
10	hsa-mir-9-3	I; II; III; IV	35	hsa-mir-122	I; II
11	hsa-mir-1-2	I	36	hsa-mir-10a	I
12	hsa-mir-7-1	I; IV	37	hsa-mir-378a	I
13	hsa-mir-181a-2	Unconfirmed	38	hsa-mir-23b	I; II; III
14	hsa-mir-218-2	I	39	hsa-mir-193a	I; II
15	hsa-mir-150	I; II; III; IV	40	hsa-mir-451a	I
16	hsa-mir-124-1	I; II	41	hsa-mir-204	I; II
17	hsa-mir-7-2	I; II; IV	42	hsa-mir-24-2	I; II
18	hsa-mir-196a-2	I	43	hsa-mir-708	I
19	hsa-mir-124-2	I; II	44	hsa-mir-18b	I
20	hsa-mir-7-3	I; IV	45	hsa-mir-196b	II
21	hsa-mir-199b	I; II; III	46	hsa-mir-103a-1	I
22	hsa-mir-19b-2	I	47	hsa-mir-135a-1	I
23	hsa-mir-181b-1	I	48	hsa-mir-135a-2	I
24	hsa-mir-15b	I; II	49	hsa-mir-137	II
25	hsa-mir-195	I; II; III	50	hsa-mir-206	II; III

I, II, III and IV represent dbDEMC, PhenomiR, miR2Disease and miRwayDB, respectively

Prostatic neoplasms is the most prevalent nonskin cancer among men worldwide and is commonly found in men over 50 years of age. Although it has an indolent course, prostate cancer remains the third-leading cause of cancer death in men [51]. In recent years, the miRNA profiling studies demonstrate that miRNAs may act independently or in partnership with other transcription factors to regulate gene transcription, which ultimately leads to perturbed cellular processes in prostate cancer [52]. For instance, it has been suggested that hsa-miR-29b could act as an antimetastatic miRNA for prostate cancer cells at multiple steps in a metastatic cascade by regulating epithelial–mesenchymal transition signaling [53]. The top 50 prostate cancer-related miRNAs predicted by our method is listed in Table 5. As a result, 49 of the top 50 predicted miRNAs were confirmed to be associated with prostate cancer by at least one database from dbDEMC, PhenomiR, miR2Disease and miRwayDB. The only unconfirmed miRNA was hsa-mir-429. Actually, studies have demonstrated that the downregulation of miR-429 inhibits cell proliferation by targeting p27Kip1 in human prostate cancer cells. Our prediction results further confirmed its association with prostate cancer.

**Table 5:** Top 50 predicted miRNAs associated with prostatic neoplasms based on known associations in HMDD

Rank	miRNA	Evidence	Rank	miRNA	Evidence
1	hsa-mir-155	I; II	26	hsa-mir-196a-2	I; II
2	hsa-mir-18a	I; II	27	hsa-mir-429	Unconfirmed
3	hsa-mir-19a	I; II	28	hsa-mir-199b	I; II; III
4	hsa-mir-19b-1	I; II; III	29	hsa-mir-181b-2	I; II; III
5	hsa-let-7a-3	I; II; III	30	hsa-mir-150	I; II
6	hsa-let-7a-2	I; II; III	31	hsa-mir-138-2	II
7	hsa-mir-29c	I; II; IV	32	hsa-mir-138-1	II
8	hsa-mir-9-1	I; II	33	hsa-mir-125a	I; II; III
9	hsa-mir-181a-1	I; II; III	34	hsa-mir-24-1	I; II; III
10	hsa-mir-210	I; II; III	35	hsa-mir-30a	I; II; III
11	hsa-mir-7-1	I; II; IV	36	hsa-mir-196a-1	I; II
12	hsa-mir-181a-2	I; II	37	hsa-mir-192	I
13	hsa-mir-9-2	I; II	38	hsa-mir-302b	I; II
14	hsa-mir-9-3	I; II	39	hsa-mir-451a	I
15	hsa-mir-10b	I; II; III	40	hsa-mir-103a-2	I
16	hsa-mir-7-2	I; II; IV	41	hsa-mir-302a	I; II; IV
17	hsa-mir-142	I; II	42	hsa-mir-20b	I
18	hsa-let-7f-2	I; II; III	43	hsa-mir-18b	I
19	hsa-let-7i	I; II	44	hsa-mir-10a	I; II; III
20	hsa-let-7e	I; II	45	hsa-mir-302c	I; II
21	hsa-let-7 g	I; II; III	46	hsa-mir-24-2	I; II; III
22	hsa-mir-7-3	I; II; IV	47	hsa-mir-137	II
23	hsa-let-7f-1	I; II; III	48	hsa-mir-206	I; II
24	hsa-mir-218-2	I; II; III	49	hsa-mir-149	I; II; III
25	hsa-mir-19b-2	I; II; III	50	hsa-mir-135a-1	I; II; IV

I, II, III and IV represent dbDEMC, PhenomiR, miR2Disease and miRwayDB, respectively

To demonstrate the applicability of our method to diseases without any known miRNAs, we carried out the second type of case studies for Breast neoplasms (Additional file 5). Breast neoplasms is a malignant tumor that forms from the uncontrolled growth of abnormal breast cells. Recent research on miRNAs has implicated that the loss of tumor suppressor miRNAs or overexpression of oncogenic miRNAs can lead to breast cancer tumorigenesis or metastasis [54]. In this case study, we first removed all 237 miRNAs that were confirmed to be associated with breast neoplasms by HMDD v2.0, and then prioritized all the 550 candidate miRNAs by our method. As shown in Table 6, 47 out of the top 50 predicted miRNAs were verified by HMDD v2.0, and all of them were further confirmed by at least one database from dbDEMC, PhenomiR, miR2Disease and miRwayDB.

**Table 6:** Top 50 predicted miRNAs associated with breast neoplasms based on known associations in HMDD

Rank	miRNA	Evidence	Rank	miRNA	Evidence
1	hsa-mir-21	HMDD; I; II; III; IV	26	hsa-mir-29a	HMDD; I; II; IV
2	hsa-mir-155	HMDD; I; II; III; IV	27	hsa-mir-92a-2	HMDD; I; II
3	hsa-mir-17	HMDD; I; II; III	28	hsa-mir-223	HMDD; I; II; IV
4	hsa-mir-20a	HMDD; I; II; III	29	hsa-mir-181a-1	HMDD; I; II; III
5	hsa-mir-125b-1	HMDD; I; II; III; IV	30	hsa-mir-29b-1	HMDD; I; II; III
6	hsa-mir-92a-1	HMDD; I; II	31	hsa-let-7b	HMDD; I; II
7	hsa-mir-18a	HMDD; I; II; III	32	hsa-mir-200c	HMDD; I; II; III
8	hsa-mir-145	HMDD; I; II; III	33	hsa-mir-29c	HMDD; I; II; III
9	hsa-mir-16-1	HMDD; I; II	34	hsa-mir-181a-2	HMDD; I; II; III
10	hsa-mir-34a	HMDD; I; II; IV	35	hsa-mir-200a	HMDD; I; II; III; IV
11	hsa-mir-19b-1	HMDD; I; II; IV	36	hsa-mir-29b-2	HMDD; I; II; III
12	hsa-mir-125b-2	HMDD; I; II; III; IV	37	hsa-let-7c	HMDD; I; II
13	hsa-mir-146a	HMDD; I; II; III; IV	38	hsa-mir-19b-2	I; II; IV
14	hsa-mir-19a	HMDD; I; II; IV	39	hsa-mir-150	I; II
15	hsa-mir-16-2	HMDD; I; II	40	hsa-mir-210	HMDD; I; II; III
16	hsa-mir-221	HMDD; I; II; III	41	hsa-mir-34c	HMDD; I
17	hsa-let-7a-1	HMDD; I; II; III	42	hsa-mir-1-1	HMDD; I; II; IV
18	hsa-mir-143	HMDD; I; II; III	43	hsa-let-7d	HMDD; I; II; III
19	hsa-let-7a-3	HMDD; I; II; III	44	hsa-mir-182	HMDD; I; II; III
20	hsa-mir-126	HMDD; I; II; III	45	hsa-mir-214	HMDD; I; II; IV
21	hsa-mir-15a	HMDD; I; II	46	hsa-mir-9-1	HMDD; I; II; III; IV
22	hsa-mir-31	HMDD; I; II; III	47	hsa-mir-106b	HMDD; I; II
23	hsa-let-7a-2	HMDD; I; II; III	48	hsa-mir-142	I; II; IV
24	hsa-mir-200b	HMDD; I; II; III; IV	49	hsa-let-7i	HMDD; I; II; III
25	hsa-mir-222	HMDD; I; II; III	50	hsa-mir-181b-1	HMDD; I; II; III; IV

I, II, III and IV represent dbDEMC, PhenomiR, miR2Disease and miRwayDB, respectively

Lastly, we conducted the third type of case studies for Hepatocellular Carcinoma in which the older version of HMDD was used to prioritize miRNAs with the given disease and the latest version of HMDD (i.e. v2.0) was adopted to evaluate the prediction results (Additional file 6). Concretely, there were 1475 known associations involving 281 miRNAs and 129 diseases recorded in the older version of HMDD. The top 50 ranked miRNAs predicted by our method were listed in Table 7. As a result, 38 out of them were confirmed by HMDD v2.0, and all of them were validated by at least one of the four databases dbDEMC, PhenomiR, miR2Disease and miRwayDB. Notably, we found that although hsa-mir-9-1, hsa-mir-132, hsa-mir-194-1 and hsa-mir-9-2 were not recorded in HMDD v2.0, they were all confirmed by the four databases, indicating their potential functional roles in the pathogenesis of Hepatocellular Carcinoma. In summary, all the three types of case studies further validated the effectiveness and reliability of our method in uncovering potential associations between miRNAs and diseases.

**Table 7:** Top 50 predicted miRNAs associated with hepatocellular carcinoma based on known associations in the older version of HMDD

Rank	miRNA	Evidence	Rank	miRNA	Evidence
1	hsa-mir-155	HMDD; I; II; III; IV	26	hsa-mir-214	HMDD; I; II; III
2	hsa-mir-16-1	HMDD; I; II; III; IV	27	hsa-mir-150	HMDD; I; II; III
3	hsa-let-7a-3	HMDD; I; II; III	28	hsa-mir-181b-2	HMDD; I; II; III
4	hsa-let-7a-2	HMDD; I; II; III	29	hsa-mir-29c	HMDD; I; II
5	hsa-mir-15a	HMDD; I; II; III	30	hsa-mir-133a-1	I; III; IV
6	hsa-let-7a-1	HMDD; I; II; III	31	hsa-mir-24-1	HMDD; I; III
7	hsa-let-7b	HMDD; I; II; III	32	hsa-mir-132	I; II; III; IV
8	hsa-mir-16-2	HMDD; I; II; III; IV	33	hsa-mir-15b	HMDD; I
9	hsa-let-7d	HMDD; I; II; III	34	hsa-mir-194-1	I; II; III; IV
10	hsa-let-7c	HMDD; I; II; III	35	hsa-mir-205	HMDD; I; III; IV
11	hsa-mir-143	I; II; III	36	hsa-mir-9-3	I; III; IV
12	hsa-let-7i	HMDD; I; II	37	hsa-mir-9-2	I; II; III; IV
13	hsa-let-7f-2	HMDD; I; II; III	38	hsa-mir-25	HMDD; I; II; III
14	hsa-mir-29b-1	HMDD; I; IV	39	hsa-mir-200c	HMDD; I; II
15	hsa-mir-181b-1	HMDD; I; II; III	40	hsa-mir-373	HMDD; I
16	hsa-mir-126	HMDD; I; II; III	41	hsa-mir-429	I; IV
17	hsa-mir-133a-2	I; III; IV	42	hsa-mir-302b	HMDD; I; II; IV
18	hsa-let-7f-1	HMDD; I; II	43	hsa-mir-339	I; II
19	hsa-mir-29a	HMDD; I	44	hsa-mir-210	HMDD; I; II
20	hsa-let-7 g	HMDD; I; II; III	45	hsa-mir-30c-1	HMDD; I; II; III
21	hsa-mir-141	HMDD; I; II; III; IV	46	hsa-mir-34b	I
22	hsa-mir-106b	HMDD; I; II; III	47	hsa-mir-206	I
23	hsa-mir-146b	HMDD; I	48	hsa-mir-181a-2	HMDD; I; II; III
24	hsa-mir-9-1	I; II; III; IV	49	hsa-mir-107	HMDD; I; III
25	hsa-mir-181a-1	HMDD; I; II; III	50	hsa-mir-196a-2	HMDD; I

I, II, III and IV represent dbDEMC, PhenomiR, miR2Disease and miRwayDB, respectively

## Discussion

The experimental results presented above clearly demonstrated the superior performance of our method. Moreover, the results of case studies on five common human diseases further confirmed the utility of the proposed method. Intriguingly, we noticed that for lung neoplasms and ovarian neoplasms, miRNAs within the same family of the unconfirmed miRNAs in the top 50 predicted miRNAs were essentially verified to be related with the investigated diseases by dbDEMC. As a matter of fact, evidence have demonstrated that miRNA family/cluster could function together in various pathological processes, such as miR-200 family, let-7 family and etc. [55, 56]. Therefore, our results provided new evidence that miR-520 family and miR-181 family might play vital roles in lung neoplasms and ovarian neoplasms, respectively.

The success of our model could be largely attributed to the following two reasons. Firstly, the $\ell_{1}$-norm imposed on our objective function could generate sparse solutions, which makes our method robust to the incompleteness of current datasets. Secondly, both of the reconstructed miRNA functional similarities as well as the disease semantic similarities could be adaptively re-weighted according to the learned label matrix during each iterations. As a result, miRNAs or diseases with higher similarities will get more similar predicted labels and vice versa. However, there are still rooms for improvements in our model. In essence, since the miRNA functional similarity matrix as well as disease semantic similarity matrix was updated separately in their own spaces, our model is expected to be more effective if we could combine the two optimization spaces in a more reasonable manner. Besides, more data sources such as miRNA sequence similarities and miRNA family information should be integrated into our model to further improve the prediction ability of our model.

## Conclusion

MiRNAs have been established as key metastasis regulators in diverse disease types. The ability of these small non-coding RNAs to regulate gene expression has generated much interests in exploiting them as potential therapeutic biomarkers in human diseases [57]. The accumulating amount of data from multiple sources have posed great opportunities in the identification of miRNA-disease associations based on computational models at a large scale. In this paper, we presented a novel semi-supervised prediction model based on $\ell_{1}$-norm graph. To alleviate the influences of the intrinsic noise existing in the current datasets, we first recalculated the miRNA functional similarities and disease semantic similarities with the latest version of Mesh descriptors and HMDD. We then introduced an effective $\ell_{1}$-norm based objective function and iteratively updated the confidence for unconfirmed miRNA-disease associations in both miRNA space and disease space. The experimental results of global LOOCV and local LOOCV intuitively demonstrated the effectiveness of the proposed method. In addition, the comparison results between our method and five state-of-the-art methods further confirmed the superior performance of our method. More importantly, our method could require a reasonable amount of computational resources to achieve comparable results. Lastly, the ability of our method in predicting potential miRNA-disease associations was verified by the three types of case studies performed on five common diseases. In summary, our method could serve as a reliable and efficient tool to detect novel associations between miRNAs and diseases.

## Additional files

**Additional file 1.** Disease semantic similarity matrix.

**Additional file 2.** MiRNA-disease association matrix.

**Additional file 3.** MiRNA functional similarity matrix.

**Additional file 4.** Results of the first type of case study on 16 common diseases.

**Additional file 5.** Results of the second type of case study on 16 common diseases.

**Additional file 6.** Results of the third type of case study on 16 common diseases.

## Abbreviations

MiRNA: microRNA
LOOCV: leave-one-out cross validation
ROC: receiver-operating characteristics curve
AUC: the area under ROC curve
